# Polyandry Depends on Postmating Time Interval in the Dengue Vector *Aedes aegypti*

**DOI:** 10.4269/ajtmh.15-0893

**Published:** 2016-04-06

**Authors:** Ethan C. Degner, Laura C. Harrington

**Affiliations:** Department of Entomology, Cornell University, Ithaca, New York

## Abstract

*Aedes aegypti* is the primary vector of the dengue and chikungunya viruses. After mating, male seminal fluid molecules cause females to become unreceptive to a subsequent mating. This response is often assumed to be immediate and complete, but a growing body of evidence suggests that some females do mate more than once. It is unknown how quickly a female becomes unreceptive to a second mating. Furthermore, the degree to which she remains monandrous after laying several batches of eggs has not been rigorously tested. Therefore, we assessed the rates of polyandry in two sets of experiments using wild-type males and those with fluorescent sperm. The first experiment tested the likelihood of polyandry after postmating intervals of various durations. Most females became refractory to a second mating within 2 hours after mating, and rates of polyandry ranged from 24% immediately after mating to 3% at 20 hours after mating. The second experiment tested whether females were polyandrous after cycles of blood meals and oviposition. No re-insemination was found after one, three, or five such cycles. This study is the first to demonstrate that polyandrous behavior depends on the postmating interval. Our results will inform future applications that depend on an accurate knowledge of *Ae. aegypti* mating behavior, including models of gene flow, investigations of molecules that drive female mating behavior, and control strategies that deploy genetically modified mosquitoes into the field.

## Introduction

The mosquito *Aedes aegypti* transmits several pathogens to humans, the most important of which are the dengue virus (DENV) and chikungunya virus. Globally, DENV causes significant disease with an estimated 96 million clinically apparent cases annually,^1^ of which 500,000 cases develop into dengue hemorrhagic fever and 22,000 are fatal.^2^ Chikungunya is an emerging viral threat and is responsible for epidemics worldwide.^3^ Although a commercial vaccine is emerging for DENV,^4^ prevention of DENV and chikungunya virus transmission still relies heavily on vector control.

Several promising approaches for mosquito control involve field releases of modified mosquitoes that must mate with the wild population. Strategies examined to date involve reduction or replacement of the vector population with mosquitoes carrying disease-refractory traits. For example, *Ae. aegypti* carrying certain strains of the endosymbiotic bacteria *Wolbachia* are resistant to DENV infection.^5^ In field trials, releases of such mosquitoes have been successful at replacing wild populations with *Wolbachia*-positive individuals.^6^ Alternatively, control strategies may deploy males that carry lethal genetic cargo to functionally sterilize females. Such releases have been tested in several countries and show promise in reducing vector populations and disease burden.^7^ Both approaches depend on successful mating interactions between released and wild mosquitoes, and thus the success of these strategies is intimately connected to mating behavior. However, some aspects of mating behavior, such as female mating frequency, remain poorly understood.

Normally, when *Ae. aegypti* females mate they become refractory to remating.^8^,^9^ This response can also be induced by injecting male seminal fluids into the hemocoel,^10^,^11^ but it is currently not known how soon the active molecule (or molecules) in seminal fluid takes effect. In *Aedes* *albopictus,* females can be re-inseminated if their second mating occurs within 40 minutes of their first mating.^12^ In the malaria vector, *Anopheles gambiae*, males prevent polyandry by transferring the hormone 20-hydroxyecdysone in their ejaculate, but this molecule also has a delayed effect, with full refractoriness not induced until 1–2 days postmating.^13^ A similar effect of seminal fluid has been well characterized in *Drosophila melanogaster*, in which refractoriness is induced by the seminal fluid protein sex peptide.^14^,^15^ Sex peptide does not immediately prevent a second mating,^16^ and females are frequently re-inseminated within 4 hours.^17^ However, other mechanisms reduce insemination in this latent period, including the seminal fluid protein PEBII^17^ and increased expression of a cuticular hydrocarbon that reduces attractiveness.^18^

In *Ae. aegypti*, polyandry has been documented in the laboratory,^8^,^19^–^21^ a semi-field enclosure,^22^ and a small-scale study of a wild population.^23^ How polyandrous behavior changes throughout a female's postmating life is poorly understood, but knowing when and why polyandry occurs has tremendous importance to population suppression strategies that deploy genetically modified males; if females remate most frequently after mating with modified males, they could undermine the success of inundative releases.^24^ Some models of release strategy outcomes do not incorporate polyandry,^25^,^26^ but understanding baseline levels of polyandry is necessary for optimal development of a predictive modeling framework. Studying polyandry will also assist in predicting the spread of genes or traits in population replacement scenarios,^27^ and it will also provide insight into what drives male and female reproductive success.

Here, we examine two essential dynamics of female mating behavior: how quickly she becomes unreceptive to a second mating and the extent to which she remains unwilling to mate over the course of her reproductive life.

## Methods

### Mosquitoes.

Two strains of *Ae. aegypti* mosquitoes were used: a Thai strain established from mosquitoes captured in Bangkok, Thailand, supplemented periodically with new field-collected individuals, and a DsRed transgenic strain with sperm that express the red fluorescent protein, DsRed, emitting red light under green (557 nm) illumination.^28^ All sperm from this strain were verified to display red fluorescence. Both strains were reared under similar conditions in a climate-controlled chamber at 28.0°C with 71.9% ± 9.5% relative humidity and a daily light regime of 10-hour light:10-hour dark with 2-hour simulated dusk and dawn. After vacuum hatching, larvae were reared to pupae at a density of 200 larvae per 1 L of deionized water. Each tray of 200 larvae received four Cichlid Gold fish food pellets (Hikari, Himeji, Japan) to produce adults of uniform size (Supplemental Methods). Females eclosed in individual test tubes to ensure virginity; males eclosed in 8-L bucket cages, with any incidental females removed before 24 hours.^29^ Adults were held in separate cages by sex at a density of 200 adults per cage with access to 10% sucrose ad libitum. All mosquitoes used were 4–7 days old.

### Virgin mating rate.

To establish a baseline mating rate using our two strains, 100 virgin Thai females were allowed to mate with DsRed males for 2 hours at a density of 10 males and 10 females per 2-L carton. After 2 hours, males were removed and mating rate was determined by checking females' reproductive tracts for fluorescence.

### Onset of refractoriness.

We tested the hypothesis that after a female's first mating, she gradually becomes refractory to a second mating over time. Females were allowed to remate at different periods after their first mating to determine how the likelihood of re-insemination changes. Eight treatments corresponded to different postmating time intervals during which females were not allowed to remate: 0–2, 2–4, 4–6, 6–8, 8–10, 10–12, 14–16, and 20–22 hours postmating (hpm). Five replicates were conducted on different days: three in which all intervals were tested and two additional days on which only the first three intervals were tested.

First, matings between Thai females and males were observed. Virgin females were introduced singly or doubly into an 8-L cage with up to 15 males. Fresh virgin males were added to cages as the numbers became depleted. The total number of males in mating cages was not held constant over time because it did not impact the individual mating interactions that we carefully monitored and captured in this study. To encourage flight, the cage was knocked gently every time a female landed without coupling. Upon coupling, male and female pairs were observed closely for firm genital union. Those males and females that locked terminalia for longer than 8 seconds were considered successfully mated. The accuracy of this method was verified in a preliminary experiment by checking the spermathecae of females after their observed matings; one female out of 100 was not inseminated. After copulation terminated, both the male and the female were removed. All males and those females that mated for shorter than 8 seconds were discarded. Inseminated females were kept in groups of 10 in a 2-L carton, and the time at which each group was inseminated was recorded. To account for the effect of temperature and humidity on mating behavior, all matings were conducted in a climate-controlled environmental chamber under the conditions described above for rearing. After the appropriate postmating interval, 10 DsRed males were added to each carton, and females were given the opportunity to remate for 2 hours. To ensure that females in the 0–2 hpm treatment were allowed to remate immediately after mating, females in this treatment were directly placed into a carton of 10 DsRed males as soon as they were mated (less than 30 seconds after mating). This procedural difference was verified to have no effect on remating frequency (Supplemental Methods). All remating periods were conducted between 1100 and 1400 hours (with dawn at 0600 hours), and the exact time at which this period began was recorded. After the remating period males were discarded.

Females receive semen into their bursa, from which sperm travel to spermathecae (long-term storage organs) within minutes.^30^ If they receive a second insemination, they may or may not store sperm from the second male. Polyandrous females were defined as those that received a second insemination into their bursa, regardless of whether the second male's sperm were stored. To determine polyandry, females were anesthetized on ice and dissected in a drop of physiological saline. The reproductive tract was removed from each female, and the bursa was examined for DsRed fluorescence; any female that had fluorescence in her bursa was scored as remated. To verify that females were successfully mated by Thai males, the spermathecae of any DsRed-mated females were broken open by gently applying pressure from a coverslip, and sperm from each female were observed for fluorescence. Females with a red bursa and any wild-type (non-red) sperm were considered polyandrous. Those with only DsRed sperm were considered to have never received a Thai male insemination, despite observation of copulation; these accounted for 1.7% of females (range = 0.6–2.9% across treatments). This proportion is consistent with the failed insemination rate (1%) observed in females after observed matings.

Females that were not successfully inseminated by a Thai male were discarded from analysis. Remating cartons of 10 females were considered the experimental unit, and the proportion of re-inseminated females per carton was square-root transformed to normalize residuals. A univariate general linear mixed model was constructed using the transformed data as the response variable. Explanatory variables included the postmating interval as a fixed factor, the time of day at which remating period occurred as a covariate, replicate as a random factor, and all factorial interactions between these terms. Although postmating interval is a temporal variable, it was modeled as a categorical variable because the experimental design included eight discrete treatments, and these intervals were not evenly spaced. The model was run iteratively; the term with the highest *P* value was removed each time until all remaining terms were significant predictors (*P* < 0.05). Post hoc pairwise comparisons were made using estimated marginal means with a Bonferroni correction and a significance threshold of *P* = 0.05. In most virgin cartons, all females mated, and therefore these data did not fit a normal distribution. Therefore, comparisons of each postmating treatment to virgins were made using one-sample *t* tests, with the null hypothesis conservatively chosen as 0.8, the lowest proportion of mated females observed in a carton. Statistics were performed using SPSS (IBM SPSS Statistics for Windows, version 21.0, Armonk, NY).

### Gonotrophic cycles.

We tested the hypothesis that females become more receptive to a second mating after completing multiple gonotrophic cycles (i.e., taking multiple blood meals and producing several batches of eggs). Females were assayed for remating after one, three, and five gonotrophic cycles. To control for the possibility that the length of the post-mating time interval, rather than progression through gonotrophic cycles, may affect a female's mating behavior, parallel treatments were included that were fed only sucrose and did not lay any eggs. Previous authors have determined that nearly all 28-day old virgin females are receptive to insemination,^20^ so a control of the mating ability of old females was not included in this study.

Virgin Thai females were mated en masse to virgin Thai males by combining 200 females and 200 males in 8-L cages for 2 days. All mosquitoes were then cold anesthetized and males were discarded. Females, now 4–5 days old, were separated into blood- and sucrose-fed treatments. Each gonotrophic cycle lasted 6 days and began with blood-fed females receiving a blood meal from ECD (Cornell Institutional Review Board human subjects activity exemption, FWA 00004513). After each feeding, females that took a blood meal were separated from those that did not. Unfed females were given another opportunity to feed several hours later, and those that still did not feed were discarded. Females were given oviposition substrate 4 days after blood feeding. A solution of 10% sucrose was provided to both treatments ad libitum, except for 24 hours before each blood meal, when it was replaced with deionized water in both treatments to facilitate feeding.

After one, three, and five gonotrophic cycles (6, 18, and 30 days, respectively), a subset of at least 90 females was removed from each treatment and placed into 2-L cartons with 4–7 days old DsRed males at a density of 10 males and 10 females. After 24 hours, males were removed and female reproductive tracts were examined for remating as described above. This experiment was replicated twice, for a minimum of 18 cartons total per treatment. Sample sizes are included in Table 1. No statistical tests were conducted on these data due to a lack of polyandry.

## Results

Postmating time interval significantly predicted remating frequency (univariate general linear mixed model; *F* = 9.031; df = 7,158; *P* < 0.001) (Supplemental Table 1). The only other significant predictor was the day on which matings were conducted (log-likelihood test; Δ*G*^2^ = 8.154; *P* = 0.004). We included this random factor in our final model to control for any potential effect of day. The estimate of residual covariance was 0.034, of which 0.006 (18%) was explained by the day effect.

**Figure 1.**
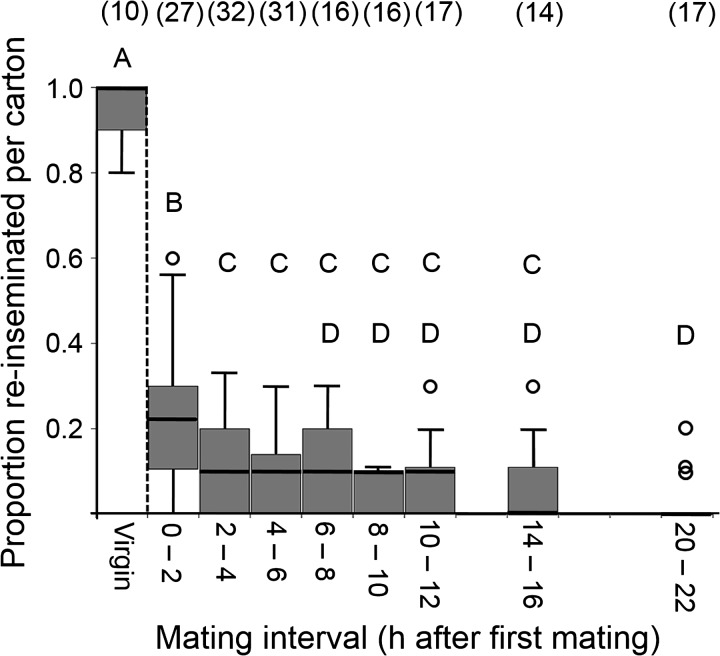
Onset of Refractoriness results. Proportion of virgin female *Ae. aegypti* mosquitoes inseminated (left of dashed line) and females re-inseminated at different post-mating intervals (right of dashed line). Virgins mated more frequently than females in all subsequent treatments (One-sample *t* test; *t* < −12.31; *P* < 0.001). Post-mating interval significantly predicts re-mating likelihood (Univariate general linear model; *F* = 9.031; *P* < 0.001). Only mated samples (right of the dashed line) were included in the model. Treatments with the same letter are not significantly different from each other (Post hoc pairwise comparisons with Bonferroni correction; *P* > 0.05). Untransformed data are shown, but analyses were performed on square root transformed data. Boxes and bold lines represent inner quartiles, whiskers are drawn using the Tukey method, and circles indicate outliers. Sample sizes (number of cartons containing 10 females) are included in parentheses.

After mating, 24% ± 3.0% (mean ± SE) of females were re-inseminated from 0 to 2 hpm. All subsequent intervals had a significantly lower rate of re-insemination than 0–2 hpm, ranging from 3.0% ± 1.4% (20–22 hpm) to 11% ± 2.6% (6–8 hpm). There was no significant difference between any intervals within 2–16 hpm. The interval with the least polyandry was 20–22 hpm (3.0% ± 1.4%); it was significantly lower than all intervals before 6 hpm, but not different from intervals after 6 hpm. Each postmating interval had a significantly lower insemination rate than virgins, of which 96% were inseminated (*t* < −12.31; *P* < 0.001) (Figure 1

).

No females that completed one, three, or five gonotrophic cycles were re-inseminated. Similarly, no sucrose-fed control females were re-inseminated, except for two questionable mosquitoes after 18-day postmating. The bursae of these females had a faint fluorescence with small fluorescent particles scattered throughout the organ. The fluorescence was atypical of females inseminated by DsRed males, but was clearly above levels of background fluorescence. No fluorescence was observed in these females' spermathecae. Because only two females had this phenotype, they were unlikely to be biologically relevant, and thus we excluded them from further discussion.

## Discussion

This study addresses two poorly understood but important aspects of *Ae. aegypti* mating behavior: the speed with which a female becomes refractory to a second mating and the degree to which this refractoriness is maintained over multiple gonotrophic cycles. Our results support recent studies demonstrating that re-insemination may occur in some situations.^22^,^23^ It is likely that reported cases of polyandry in these studies occurred before 22 hpm. In addition, we show that females do not mate after 6-day postmating, regardless of progression through up to five gonotrophic cycles. Although the age of field-collected individuals is difficult to estimate, the majority of females likely do not survive beyond five gonotrophic cycles.^31^ Therefore, we are confident that we encompassed the reproductive lifespan of female *Ae. aegypti* in our study.

Most females (76%) became refractory to a second mating within 2 hours of their first mating. This behavioral shift may have occurred sooner, but we did not test earlier intervals. Females' shift to refractoriness undoubtedly benefits males by limiting the opportunity for sperm competition,^32^ but the outcome for females is less certain. A polyandrous female may risk acquiring venereal pathogens,^33^ or receiving an increased volume of seminal fluid may shorten her lifespan.^34^ On the other hand, a female may benefit by receiving nutrition in the ejaculate^35^,^36^ or extra sperm if the first insemination was insufficient.^19^ It is possible that females use an appraisal of their first male (based on his ejaculate, behavior, size, or other phenotype) to decide whether to mate again. Instances of skewing postmating promiscuity based on first male cues have been documented in other species, from solitary bees^37^ to hamsters.^38^,^39^ If this is the case, a female may reap genetic benefits by increasing the proportion of her offspring that are sired by a second, more suitable mate.^40^ Although we did not test the proportion of a female's offspring sired by the first and second males, other authors have documented mixed-paternity offspring from females.^8^,^23^ Future investigations should quantify how multiple insemination affects first and second male reproductive success. Whether a female ultimately remates is likely an integration of male manipulation and female interests and probably contributes to sexual conflict.

In our study, more females were refractory at 2–4 hpm (89%) than at 0–2 hpm, but the level of polyandry remained stable in intervals from 2 to16 hpm (5.8–11%). We expected a steady decline in receptivity and were surprised to observe a static number of unreceptive females during the 2–16 hpm period. We hypothesize that refractoriness is induced in two steps: most females lose their willingness to remate shortly after their first mating and the remaining receptive females become refractory gradually sometime after 16 hpm. Whether the mechanisms that elicit this two-step response are the same or different remains unknown, but we caution against the assumption that all of a female's postmating receptivity is dictated by a single mechanism. In *D. melanogaster*, before sex peptide induces long-term refractoriness, other molecules prevent females from remating.^14^,^15^ These include the seminal fluid protein PEBII^17^ and a male-transferred pheromone that reduces female attractiveness.^18^ Similarly, although long-term refractoriness is induced in *Ae. aegypti* by a seminal molecule (or molecules),^11^,^41^,^42^ females may not be under strict control of this molecule before 16 hpm.

In the onset of refractoriness experiment, females mated most infrequently (3.0%) at 20–22 hpm, the last time interval we tested. We hypothesize that complete refractoriness sets in shortly thereafter. Helinski and others^11^ found that no females injected with male accessory gland extract were inseminated 2 days after injection, but they did not test earlier time points. Even if re-insemination were to occur at a similarly low rate after 22 hpm, polyandry at this time is unlikely to be biologically relevant. Females typically mate in flight near the host^43^ and are thus likely to feed soon after their first mating. Therefore, their mating opportunities would be limited while they rest, digesting their blood meal.

We found that once female refractoriness is established, it is complete, long lasting, and independent of reproductive status; no females that completed up to five gonotrophic cycles were re-inseminated despite having the opportunity to remate, nor were sucrose-fed controls. This result agrees with Helinski and others,^11^ who found that no re-insemination occurred in sucrose-fed females up to 34 days after injection of male accessory gland extract. A similar result was found in *An. gambiae*, which also failed to remate after five gonotrophic cycles.^44^ Female re-insemination in these disease vectors may be futile because irreversible postmating changes in reproductive tract structure may prevent a female from storing a second male's sperm.^45^ Furthermore, most *Ae. aegypti* females store a lifetime's worth of sperm from their first insemination,^46^ and *Ae. albopictus* females do not suffer reduced fertility after six gonotrophic cycles.^12^ Therefore, selective pressure for the ability to replenish sperm is probably weak.

Our results contrast with two reports that polyandry after multiple gonotrophic cycles is common in *Ae. aegypti*.^20^,^21^ The source of this discrepancy is uncertain but may lie in methodological differences. While we directly observed transferred semen from a second male, previous studies used the amount of seminal fluid in the bursa^20^ or the transfer of radioactive isotopes from males to females^21^ as indirect proxies for re-insemination. Another source of variation could be the strain of *Ae. aegypti* used. Both studies that found re-insemination used the Rockefeller strain of *Ae. aegypti*, which has been maintained in laboratory colonies since the 1930s or earlier,^47^ while we used mosquitoes derived from a recent field collection in Thailand. Laboratory evolution of polyandry has been documented in another insect,^48^ and laboratory-adapted strain history could contribute to the differing results between previous studies and ours.

The experimental conditions in this study did not replicate the opportunities for remating that females experience in the field. However, carefully controlled studies like ours cannot be conducted in the field. In the wild, males typically intercept females as they approach a host to feed,^43^ and thus female exposure to males is likely limited to when females are host seeking. In our experiments, spatial constraints and duration of exposure to second males may have artificially inflated rates of polyandry. On the other hand, lack of host cues and actual opportunity to feed may have discouraged polyandry. Furthermore, the DsRed males used to assess polyandry were not as competitive for mates as their Thai counterparts (Supplemental Methods). Therefore, whether our experiments overestimate or underestimate remating is uncertain. Nonetheless, rates of polyandry in our study are similar to those found in a study conducted in a semi-field enclosure (14%)^22^ and in a small-scale study of wild-caught females (6.25–14.6%).^23^ Knowing the frequency, timing, and ecological context of male–female encounters in a natural setting would aid our understanding of polyandry in the field.

While absolute frequencies of polyandry in our study may not be generalizable to all field populations, we nonetheless have described for the first time the dynamics with which refractory behavior is induced after mating and maintained over a female's postmating life. Our novel results will inform investigations aiming to identify molecules and pathways responsible for female postmating behavior. In addition, understanding female mating frequency is an important consideration for vector-control strategies that deploy genetically modified males or *Wolbachia*-infected females.^49^ Poor male quality or strain incompatibility might encourage females to mate again, and this could allow females to partially circumvent vector control efforts by mating with a second, wild-type male. Increasing the number of released mosquitoes could compensate for the effect of nonrandom mating, and thus knowing how polyandry influences vector control outcomes is necessary for calculating how many mosquitoes to release. Therefore, this laboratory study provides a foundational understanding of when polyandry is likely to occur in *Ae. aegypti*, and our results will guide future assessments of remating frequency between strains relevant to specific release programs.

## Supplementary Material

Supplemental Datas.

## Figures and Tables

**Table 1:** Number of females tested in each gonotrophic cycle treatment (GC) and its corresponding sucrose-fed control (S)

	Replicate A	Replicate B	Total
1 GC	88	83	171
1 S	96	85	181
3 GC	83	98	181
3 S	99	91	190
5 GC	148	130	278
5 S	137	93	230
