# Supplementary material for: Polyandry Depends on Postmating Time Interval in the Dengue Vector Aedes aegypti
Source: Am J Trop Med Hyg. 2016 Apr 6;94(4):780–5. doi: 10.4269/ajtmh.15-0893 (PMC4824218; doi:10.4269/ajtmh.15-0893)
Supplement: Supplementary file 1 [file SD2.pdf]

SUPPLEMENTAL METHODS

Mosquito size determination. A pilot experiment was replicated twice to verify that our rearing conditions and feeding regimen produced females of uniform size. One wing was taken from 29 males of each strain from each replicate, mounted on double sided tape, and measured from the ancillary incision to the distal fringe. In our subsample, mean wing lengths ranged from 2.2 to 2.3 mm with a maximum standard error of 0.01 mm. DsRed males' wings were 1.8–2.3% longer than wild-type males.

Testing the effect of male addition method for 0–2 hpm. For the 0–2 hpm interval, the method of adding females to cartons of DsRed males differed from all later time intervals. In this interval, females were added one-by-one to a carton of 10 DsRed males immediately after the female's first mating, whereas in later intervals, 10 DsRed males were added en masse to a carton of 10 mated females. This was done to ensure that each female in the 0–2 hpm interval had the opportunity to mate immediately, as a time lag for one-by-one observations was approximately 10 minutes per carton. To verify that this procedural difference did not alter the mating rate of females at 0–2 hpm, we compared the two methods directly for the 0–2-hpm time period, and found no significant difference between the two intervals (Mann–Whitney test; $N_{1,2} = 14$; $Z = 0.689$; $P = 0.94$).

Direct competition assay of Thai and DsRed males. To assess the potential for differential mating success of the DsRed males and Thai males, a direct competition assay was conducted. Five virgin DsRed males, five virgin Thai males, and five virgin Thai females were allowed to mate in 2 L wax cartons for 22 hours ($N = 20$ cartons). After a day, the strain of each female's mate was determined by observing the reproductive tract for fluorescence. All females were mated, but significantly fewer (20%) were inseminated by DsRed males (Z-test, $Z = 5.94$; $P < 0.001$).

Supplemental Table 1

Individual parameter estimates of univariate general linear mixed model

Parameter	Estimate	95% CI	t	P
Intercept	0.033	−0.091–0.156	0.556	0.586
0–2 hpm	0.428	0.310–0.546	7.161	0.000
2–4 hpm	0.218	0.105–0.332	3.807	0.000
4–6 hpm	0.190	0.076–0.305	3.293	0.001
6–8 hpm	0.176	0.048–0.303	2.720	0.007
8–10 hpm	0.097	−0.030–0.226	1.516	0.132
10–12 hpm	0.179	0.053–0.304	2.809	0.006
14–16 hpm	0.082	−0.050–0.215	1.227	0.222
20–22 hpm	0	N/A	N/A	N/A

hpm = hours postmating; CI = confidence interval; N/A = not applicable. Because postmating interval was coded as a fixed (categorical) variable (see Methods: Onset of Refractoriness), our model contains nine terms: one for each postmating interval and an intercept. Each parameter is a coefficient that represents the square-root transformed data of one postmating interval. The final category, 20–22 hpm, was included as the reference level of the model, and the intercept functions as an adjustment applied to all model terms.
